# A gendered analysis of living with HIV/AIDS in the Eastern Region of Ghana

**DOI:** 10.1186/s12889-020-08702-9

**Published:** 2020-05-24

**Authors:** Adobea Yaa Owusu

**Affiliations:** Institute of Statistical, Social and Economic Research, University of Ghana – Legon, P. O. Box LG 74, Legon, Ghana

**Keywords:** HIV/AIDS, Gender differentials, Qualitative research, Health status, Healthcare, Comorbidities, Stigma, Discrimination and abuse, Lower Manya Krobo municipality, Ghana

## Abstract

**Background:**

A gender gap exists in knowledge regarding persons living with HIV/AIDS in Ghana. Women living with HIV/AIDS (WLHIV) greatly outnumber males living with HIV/AIDS (MLHIV) in Ghana and Sub-Saharan Africa generally. This necessitates more gender-nuanced evidence-based information on HIV/AIDS to guide individuals, healthcare workers, and other stakeholders in Ghana particularly. This paper undertook a gender-focused analysis of the experiences of WLHIV and MLHIV in a municipal area in Ghana which has been most impacted by HIV/AIDS.

**Methods:**

In-depth interviews of 38 HIV-positive persons recruited using combined purposive and random sampling for one month, were tape recorded and analyzed using thematic content analysis. Participants were out-patients who were receiving routine care for co-morbidities at two specially equipped HIV/AIDS Voluntary Counseling and Testing Centers in the Lower Manya Krobo Municipality (LMKM), Eastern Region, Ghana.

**Results:**

Our data yielded three major themes: characteristics of participants, health status and health seeking behavior, and challenges encountered living with HIV/AIDS. Except for feeling of sadness due to their HIV/AIDS-positive status, there were significant differences in the experiences of MLHIV, compared to WLHIV. WLHIV were more likely to be housing insecure, unemployed due mostly to stigmatization/self-stigmatization, less likely to have revealed their HIV-positive status to multiple family members, and had more profound challenges regarding their healthcare. Most MLHIV expected, demanded, and had support from their wives; WLHIV were mostly single—never married, divorced or widowed (mostly due to HIV/AIDS). The vast majority of WLHIV complained of near-abject poverty, including for most of them, lack of food for taking their anti-retroviral medicines and/or taking it on time.

**Conclusions:**

The experiences of the MLHIV and WLHIV with living and coping with the virus mostly differed. These experiences were unequivocally shaped by differential socio-cultural tenets and gendered nuances; WLHIV had more negative experiences. Public education on the extra burden of HIV/AIDS on WLHIV, more social support, and affirmative action in policy decisions in favor of WLHIV in the study district are needed to seek public sympathy and improve health outcomes and livelihoods of WLHIV particularly. Further studies using multiple sites to explore these differences are warranted.

## Background

An estimated 37.9 million [32.7–44.0 million] people were living with HIV/AIDS globally, at the end of 2018 [1, 2]. Of these, more than two-thirds (69%) were living in sub-Saharan Africa (SSA) [2, 3]. This translates into 19.4 million [17.5–21.1] persons living with HIV/AIDS in the sub-region [3]. Furthermore, despite the substantial progress in administering anti-retroviral therapy, 74% of the 1.5 million AIDS-related deaths in 2013 occurred in SSA [4]. Additionally, two-thirds of the estimated 6000 new daily HIV infections occur in SSA [4].

In terms of gender, globally, women have been the most affected by HIV/AIDS [4, 5]. It is estimated that a young woman gets infected with HIV every minute [6]. The situation has been most devastating in SSA [4, 5], where females currently comprise at least 56–59% of PLWHAs [7, 8]. Currently, 75% of new infections in SSA among young persons aged 15–19 are among girls. Additionally, women aged 15–24 years in the sub-region are twice more likely to be living with HIV compared to males in the same age bracket in the sub-region [3]. In 2017, 79% of HIV incidence in southern and eastern Africa were among adolescent females 10–19 years old [9]. In Ghana, females comprised 65% of the 334,713 PLWHAs estimated in 2018; men formed 35% [3]. HIV prevalence by sex ranged from 0.68 to 11.5% in Liberia (*P =* 0.008) in 2005 and Swaziland (*P* < 0.001) in 2006/2007 [7]. This gender disparity in HIV/AIDS prevalence in SSA starts at rather early ages [5, 7].

Sexual and gender inequality has implications for health [10], and is a very strong socio-economic filter. Regarding PLWHAs, a lot of gender nuances, both subtle and overt, have been captured by previous literature. Women’s relative vulnerability compared to men’s, is clear regarding living and coping with HIV/AIDS [11, 12]. In SSA particularly, WLHIV’s are known to have worse housing vulnerabilities, be unemployed, have financial stressors and limited social support compared to MLHIV [13–16]. Additionally, WLHIV have more intimate partner related difficulties [5, 17], stigma, abuse and discrimination [5, 18], as well as livelihood difficulties [19, 20].

Women’s disproportionate infection and impact by HIV/AIDS is attributed to women’s unequal cultural and socio-economic status which make them more vulnerable, compared to men [11, 12]. Gender norms in the sub-region propound the already known extreme vulnerability of PLWHAs and translates being WLHIV into hyper vulnerability [18, 21].

### HIV/AIDS in Ghana

In Ghana, although the prevalence of HIV reduced from 2.4 in 2016 to 2.1% in 2017, HIV incidence increased by 36% to 1.5% from 1.1% during that same period [22]. Using the 2003 Ghana Demographic and Health Survey which is nationally representative, a disproportionate HIV/AIDS gender prevalence of 1.08 was found for women [7]. Based on these data, the importance of the differential distributions of HIV/AIDS risk factors between men and women has been highlighted [7]. Age at first sex explained 92% (*p* < 0.001) of Ghanaian women’s higher HIV/AIDS prevalence. For example, a higher proportion of boys aged 15–24 years (24%) were virgins compared to females (15%). Additionally, results showed that sexual behaviors among men and women explained 47.1% of the excess HIV prevalence among women in Ghana in 2003. It was also found that the differential distribution of marital status, especially being widowed, divorced or separated accounted for 38.6% of excess HIV/AIDS infection in women in Ghana [7]. Additionally, the differential distribution of premarital sex between males and females (54.3 and 43.1%, respectively) accounted for a lower gender gap in HIV/AIDS prevalence.

Despite the gendered HIV infection in Ghana [22, 23], there is paucity of literature that focuses on the well-being of MLHIV vis-a-vis WLHIV in Ghana particularly. Furthermore, few studies have primarily focused on the intersectionality between gender and the known challenging life of being HIV-positive [19]. This paper undertakes a gendered analysis of some of the experiences of living with HIV/AIDS in the Lower Manya Krobo Municipality (LMKM). LMKM is Ghana’s most affected HIV/AIDS district [16, 23, 24]. It examines both the similarities and differences in the experiences of PLWHAs in the study district. It discusses the plausible socio-cultural factors that underpin these differences. Pre-empted by a prior review of the data [23, 25], the paper focuses on the gender differences in three thematic areas in the data: 1) socio-demographic characteristics and living conditions, 2) health status and healthcare seeking behavior, and 3) challenges with living with HIV/AIDS.

## Methods

### Study setting

**Fig. 1:**
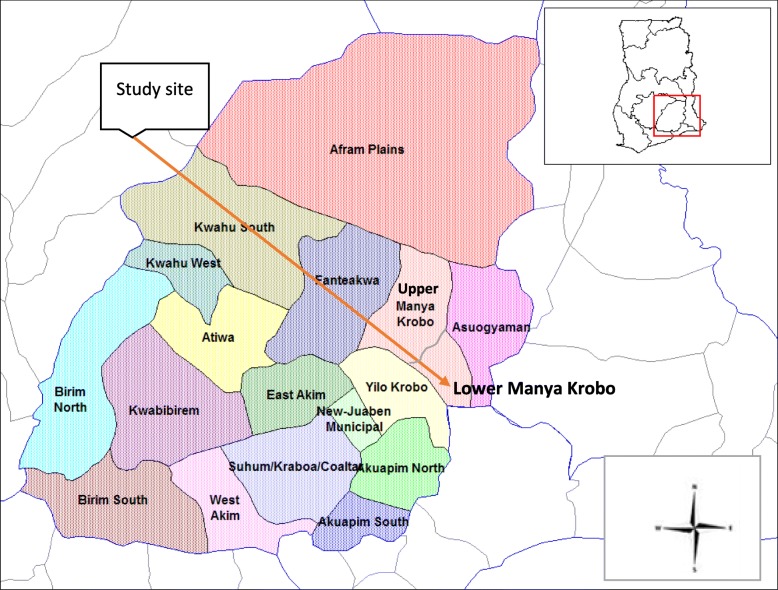
Map of the Eastern Region of Ghana highlighting the Lower Manya Krobo Municipality. Source: Adapted from Wikipedia. 2019. Wikimedia Foundation, Inc., 2019, 24 August. Author highlighted the Lower Manya Krobo Municipality with an arrow, and also inserted the arrows showing directions (north, south, east and west). https://en.wikipedia.org/wiki/Lower_Manya_Krobo_District. Accessed 27 Mar 2020

The Lower Manya Krobo Municipality (LMKM) is situated at the southeastern part of Ghana, within the Eastern Region of Ghana (Fig. 1). It formed part of the 26 administrative districts in the Eastern Region by the time of data collection. By the time of data collection, the LMKM had a total land mass of 304.4 km squared, and covered 12.4% of the Eastern Region [25, 26]. The predominant religion in the LMKM is Christianity with 92.8% adherents. Other religions are Islam and traditional African religion. The residents of LMKM are mostly farmers and fisher folks. They are indigenous Dangmes, and speak Krobo. They are patrilineal (i.e., property is inherited through the male’s lineage/fathers), and males have higher social standing traditionally, compared to females [27, 28].

Agormanya, located in the LMKM, is one of 40 HIV sentinel study sites in Ghana. The site has nearly always had the highest HIV infection rates and contributed the highest HIV/AIDS morbidity and mortality rates in Ghana since the disease was identified in the country in 1986 [23, 29] . The 2016 sentinel site prevalence ranged from 4.2% in Agormanya (which tied with Sunyani) to 0.4% in Nalerigu, with a national average of 2.4% [30].

### Study design and sampling

The research used qualitative analytical design [25, 31], which employed collecting primary field data using face-to-face approach, and adopted the grounded theory approach. Grounded theory allows employing multiple cases and viewpoints of participants, multiple crosschecking of data before framing interpretations, through constant comparison of incidents, and systematic coding of data to ensure valid and reliable analysis ([31], pp. 336–337; [32]).

The LMKM was purposively selected for the study based on the history of HIV/AIDS and the infection rates in the municipality. The municipality had two public and one mission hospitals by the time of data collection. Two of them: the St. Martin’s Depores Hospital in Agormanya and the Atua Government Hospital in Atua, were purposively selected. This is because due to the burden of infection in the LMKM, the Government of Ghana has specially equipped these two hospitals since 2002, for testing and counselling, treatment, and referral of HIV/AIDS infected persons in the LMKM and surrounding districts [14–16]. The two hospitals have stand-alone Voluntary Counselling and Testing (VCT) centers. PLWHAs prefer specialized HIV/AIDS healthcare units and utilize such facilities regularly than general healthcare facilities [33].

The participants had previously been clinically diagnosed with HIV, and self-confirmed this individually to the Principal Investigators (PIs) before they were interviewed. All of them were in the clinic for HIV/AIDS-related healthcare. They were sampled only on weekdays because the HIV/AIDS wings of the hospitals do not work on weekends. Separate sampling was done for each hospital used. Our prior recognizance visits guided us in targeting 40 HIV-positive persons at each hospital per day. We randomly selected a third of them on days there were 40 or more of them at each hospital, and on days they were less, we selected half of them into a daily pool. We hoped that these numbers would enable us spread respondent selection over the one-month data collection period. When the pool for the day had less than one-fourth males, we repeated the process to randomly pick every fourth male not selected prior. We based this on the lower male versus female PLWHAs rates in SSA in general, in Ghana as stated above, and the study district [23, 30].

Beginning with a random start, participants for the qualitative aspect of the data collection for a larger project were selected from the daily pool by one assigned qualitative interviewer among four other assigned persons who interviewed respondents for a survey aspect of the larger project. The project explored the nexus between housing and health of the PLWHAs in the LMKM. Interviewing continued till the end of each day’s clinic session. Participants who were sampled were given unique identification codes to prevent them from being re-sampled during the course of the study.

The data collection took place in June and July 2015, with the aid of a pretested in-depth interview guide using similar participants from both hospitals who were not re-interviewed in the main data collection. We sought ethical clearance and permissions from several agencies: the Ethical Review Board of the Memorial University at St. Johns, Newfoundland, Canada, the Ethics Committee for Humanities at the University of Ghana, Legon (ECH 017/14–15), and the Ghana Health Service’ Ethical Review Board (GHS-ERC: 02/11/14). Additionally, we received permissions from the Eastern Regional and LMKM Directorates of the Ghana Health Service, as well as the Administrators of the two hospitals used in the study.

The Research Assistants (RAs) were trained for three days in the objectives of the study and several techniques of field data collection, to boost the quality of our data. Two of the RAs previously worked at the hospitals where the research was conducted. The PIs supervised the field data collection, together with the retiree RAs who served as daily supervisors. The RAs were mostly Krobos and the rest had some working knowledge of Krobo. Also, all of them speak Twi (the primary local language in Ghana) and English (the official language due to Ghana’s history as a former British colony) fluently. The interviews were mainly conducted in Krobo (*n* = 32), Twi (*n* = 4) and English (*n* = 2).

The objectives of the study were explained to the participants. They were further informed that participation was voluntary, and opting out prior to the commencement or in the course of the study would attract no penalty. Only one person opted out, citing time constraints. Participants gave written consent for the interviews, tape recording, and publishing of the results, if they were literate. Otherwise, they gave verbal consent. Those who participated were assured of confidentiality. To further protect the identity of the participants, no markers about them were recorded anywhere. Participants were not given any tangible rewards although the two nurses collected their medications at each hospital’s pharmacy for them, to compensate for their time. The interviews lasted 35 to 50 min each, and were conducted in each respondent’s preferred language.

### Data analysis

The data were transcribed verbatim by three RAs, after which it was read and reviewed by the author for accuracy before the transcription was finalized. Two experts in coding of qualitative data were assigned to code the data for this paper systematically and independently, with the aid of NVivo version 11 professional software [34]. The codes were critically reviewed by a team, including the author. Subsequently, emerging themes and sub-themes were synthesized independently by two teams, and reviewed for consistency by the author. This was followed by discussions between the two coding experts, PIs and the lead RA who is an indigene of the research district and polyglot, to further interrogate the context and meaning of the data. The findings were then finalized using thematic content analysis which allows exploring all the narratives to examine how they paint the overall picture of the data [23, 35]. Based on the deductive approach, themes and sub-themes were teased out from the above-stated three sub-topics. Respondent numbers are used to support verbatim quotations in the text.

## Results

### Characteristics of participants

#### Summary characteristics

The participants were 32 females and six males. On average, they were aged 48 years (range = 25–68 years), with the exception of one woman who could not specify her age. They mostly had low socio-economic status (SES), especially the females. Six participants did not have any formal education, and only two participants had been educated to the tertiary level. The remaining participants had dropped out of school after three years of primary level education. Nearly half (18/38) of the participants had cohabited and never married formally. Six each were widowed or currently married. All participants professed heterosexual orientation. Twenty-seven participants were indigenous Ga-Dangme, and three each were Akans and Ewes. On average, they had been diagnosed HIV-positive for 6 years (range = six months to 13 years). Fifteen of them had been diagnosed between one and five years.

### Family life and family relationships

The MLHIV seemed to have more family support and connectivity, both at the nuclear and extended family levels.

*“I live alone in my room but there are other people in the house. I will like to live with my children and husband and not live alone because anything at all can happen to me at night. So if I were to be living with my children who understand my situation, I believe they can help me. I even sometimes feel lonely because I have seven children with my husband but not even one of them lives with me. This worries me very much.”* (R2, WLHIV).

Most of the MLHIV (two-thirds) were married, and two were dating, one of whom was a widower. Conversely, few of the females were married (2/32), or cohabiting (4/32). The WLHIV were mostly separated, divorced or never married. Only WLHIV were divorced, separated or cohabiting. Twelve (out of 32) of the WLHIV were widowed while only one out of six MLHIV was widowed. Seven out of 32 WLHIV were never married while one out of six MLHIV was never married.

All five MLHIVs who said their wives and siblings know of their HIV-positive status said they treat them well. Nevertheless, one MLHIV said his wife had been nagging him a lot for being unfaithful in the marriage and infecting her with HIV. Thus, all the married MLHIV said their spouses live cordially with and are supportive of them, despite knowing their HIV-positive status. Three out of four married MLHIV said their wives were HIV-negative yet they were living cordially with and are supportive of them.

*“Yes, Aunty Bea* [Nurse] *counselled us and then she* [wife] *actually realized she could cope with the situation. My wife is understandable, she understands things. And I also brought her for testing. So that’s it. She relates to me very well.”* (R18).

Even the MLHIV who said his HIV-positive wife had taken to blaming him, as a result of which she refuses to help him on the family’s farms, said she still supports him with his healthcare:

*“I’d say my wife is my family. She tells me to wake up and eat and come to the hospital****…***. *for example this morning she woke me up and told me it was time so I should come and eat and take my medicine.”* (R36).

The same applied to the six WLHIV who were married or co-habiting at the time of data collection. All of these said their spouses/partners and children live cordially with them and were supportive of them. These included the only WLHIV who was married to an HIV-negative husband, and the only WLHIV who was co-habiting who said her partner’s HIV-status was unknown at the time of data collection, because he refuses to test for it. The remaining three out of four WLHIV who were co-habiting at the time of data collection had HIV-positive partners.

*“When coming to the hospital my husband gives me money … Normal support--we have to by all means cook, so he gives us ‘chop money’. It’s normal, not anything extraordinary.” (R15, WLHIV, married).*

*“Only my husband and I know about my situation. We understand each other so we take the drugs together. My husband and children relate to me very well. Other family members don’t know about my situation so they treat me normally as they used to. My husband is also HIV-positive.”* (R6, cohabiting).

However, two WLHIV said their husbands with HIV-negative and unknown HIV-status, respectively, divorced them after disclosing their HIV-positive status:

*“I always feel lonely. I feel nobody loves me and feel I have been abandoned because I am HIV-positive. I know that was the reason why my husband sacked me from the house. If I were living like this before the disease I won’t have any problems but because I was abandoned by my husband to stay in this house because of this disease … My husband sacked me from his house after he realized I was HIV-positive and he was not. He threatened my children that if they don’t pack my things out of the house and bring them to me he was not going to feed them. I have been staying alone for about twelve years now.”* (R2).

*“I was staying with my husband when I fell sick and he said he was no longer going to marry me because I was going to die. So I left and went to stay with my mother’s relatives. Because he said I should die, he sometimes refused to give me food so I told my mother’s relatives and they came for me. My husband sacked me from the house because I am HIV positive. I was sick one day and came to the hospital and the nurses ran a test on me and said I was HIV-positive. They asked me to bring my husband so that a test can be run on him too but when he came he was HIV-negative and the nurses asked him to come after three months but he never came back. After that my husband’s attitude changed towards me and some few weeks after he sacked me from the house. After I left, he asked my children to bring my belongings to me and if they don’t he won’t give them food. I married this man when I was very young. I had all my children with him and we suffered together to put up a house and now he has sacked me. It is very painful.”* (R26).

Very few of the WLHIV said their siblings, children and other extended family members who know their HIV-positive serostatus relate to them well. Nevertheless, some WLHIV had cordial family relationships:

*“My husband’s relatives who know I have this disease relate to me very well. Some of them buy things for me. They have not maltreated me in anyway.”* (R3, WLHIV).

The WLHIV were far more likely to have very young children and/or young extended family dependants, and to be mostly raising them single-handedly. However, two MLHIV also had a fair number of young dependents. One of these MLHIV had eight biological dependents and five other dependents (mostly biological grandchildren) aged between 13 and 25 years. He said the young dependents were a source of distress to him. The other MLHIV had four biological dependents and three non-biological dependents. The latter were aged one and nine years old.

*“Now the responsibility of taking care of the children is solely dependent on me since their Mom is no more so I need someone to take care of the children for me …*” (R29, MLHIV, widower).

Participants with dependent children, mostly WLHIV, worried about the well-being of their children vis-à-vis the possible implications of their HIV-positive status.

*“Yes, I think a lot. There are times I feel physically weak then I will be thinking whether I will die and leave my little children behind. And if I leave them behind who will take care of them for me. I think of it most of the time and there are times I even cry, sometimes Iam not even able to sleep the whole night. I wake up and cry the whole time till day break, I won’t sleep.* [Do you feel depressed?] *Yes. If you think too deep/worry too much you become depressed.”* (R22, WLHIV).

*“All I fear is my children, that I don’t transmit it to them.”* (R13).

*“No,* [will not be happy if her daughter aged less than one year is diagnosed HIV-positive]. *I have prayed and wept over it, so I am just waiting on/looking up to God. I am afraid when I test the blood of my child in the lab, they might tell me she is also having the virus.”* (R25).

Some/Few participants still worried about the well-being of their adult children:

*“My son finished learning a trade. It’s been five years now but I have been unable to open a shop for him. He started working at a gold mine but does not bring anything home … When I lie down I’m unable to sleep. The boy is struggling. He should be about thirty-eight years now.” (R31, WLHIV).*

#### Disclosure/non-disclosure of participants’ HIV-positive status

Regarding disclosure, five main patterns were found with respect to the gendered nuances. First, the participants’ attempts at hiding their HIV-positive status was mostly gender-neutral. All of them but two WLHIV were working hard on hiding their HIV-positive status.

“*As for me I don’t hide anything. Most of them know I have this disease because I became very lean and because I am a little bit popular, it* [news of her infection] *spread very fast. Some of them laugh at me when I pass by them but I don’t mind them. I just pass. Some of those who mocked at me are dead because they had it but they didn’t know.”* (R5).

“*I don’t hide. All my family members know I have this disease … the days when I would suffer stigmatization are gone. Even before the family decides to do something, I would have to be present.”* (R31).

Second, whom they disclosed their HIV-positive status to showed a gendered pattern. Third, disclosure to stable romantic partners (married to or co-habiting with) was gender neutral. Fourth, disclosure to siblings showed a somewhat mixed pattern. Fifth, disclosure to adult biological children was gendered.

All of the participants, except one MLHIV, had disclosed to at least one person. Usually, this was the person who accompanied them for the initial diagnosis when they were too sick to go alone to the hospital, or when they were not responding to treatment and healthcare workers had asked them to bring someone who would support them adhere to prescribed treatment. All married PLWHAs had disclosed to their spouses. The same applied to all who were cohabiting. Although not required, healthcare workers encourage PLWHAs to come to the disclosure session with a confidant, for subsequent support, including adherence to clinic appointments and antiretroviral [36]. For MLHIV, the next most frequent confidant they had disclosed to apart from their wives was a female sibling.

*“I was diagnosed with this virus by the doctors. But I have not told anybody of my status … Because during the counselling they told us to bring somebody who loves us--like your wife; someone who could know your secret. So, because my wife was dead, I brought my sister. She is also HIV-positive. She is the one who was with me when I was admitted at the hospital … and she got to know my status … So when we started the medications, I also got to know her* (HIV-positive) *status.”* (R29).

Contrarily, the WLHIV had typically disclosed to a female sibling, or in few cases, their mother, followed in rare cases by a second female sibling, and then followed by an adult biological child. The WLHIV were far less likely to have disclosed to more than one person, and no WLHIV had disclosed to a male sibling. The WLHIV were also more likely to have told a sister who is also HIV-positive, compared to those who were HIV-negative.

*“My sister also has this disease and we understand each other. We support each other in everything. She even supports me financially because she has a drinking spot which fetches her some income. I don’t have any problem at all with it. No one knows we have this disease. It is just both of us who know. I am very happy about the fact that she supports me.”* (R4, WLHIV).

The few WLHIV who had disclosed their status to their adult sons/daughters did so in instances where they critically needed support from the latter; no MLHIV had disclosed their status to their child/children. Likewise, no respondent had disclosed their HIV-positive status to their under-aged (less than 18 years-old) child (ren).

*“I have been treating the sickness for over a year but the symptoms recur after I have seen the doctor. So with the infection breaking out on my skin it was decided that I should come with one of my children for my medication.”* (R35, WLHIV)*.*

*“I have not told my children. Still I haven’t, but my wife told one of our children, the one who accompanied her to collect her medication.* [Interviewer: So one of the children knows their mother’s status but you haven’t disclosed yours?] *Yes.* [But your wife knows you are positive?] *Yes.”* (R36, MLHIV).

### Health status and health seeking behavior

#### Physical health

Gender differences were observed in the reported physical health of the WLHIV versus the MLHIV. The MLHIV self-reported lesser body weaknesses and much fewer co-morbidities compared to the WLHIV. The most common comorbidity mentioned was diarrhoea, followed by malaria. The rest included cough, chest pains, bodily pains, skin rashes, and dizziness. Others were rheumatism, rashes, catarrh, stomach ache, and watery eyes. Ailments such as high blood pressure, diabetes, asthma, and difficulty with breathing were also mentioned.

*“I can’t walk a distance without disgracing myself. I sometimes have a problem with my stomach--running stomach, and before I walk a short distance I would have eased on myself. It’s disgraceful.”* (R23, WLHIV).

*“Sometimes before I could get up to go to the toilet I would defecate on myself.”* (R31).

Nearly all the participants also complained of the current or past side effects of the ARV. These were mostly WLHIV:

*“The drug is like ‘chop* [eat] *and sleep’ so sometimes I even sleep right after eating even before I wash my hands. So I eat on the bed so if I finish eating I just sleep on the bed and before I realize it is morning.”* (R23, WLHIV).

*“But that drug is very strong and makes me feel dizzy; this particular drug makes you feel dizzy so you can’t do anything after taking it. So I tell my child I have to sleep after taking the drug so right after eating I go to sleep.”* (R21, WLHIV).

*“As for that I was told I will feel dizzy after taking it. I didn’t know initially so I was taking it in the morning but when I complained to them they told me I shouldn’t take it in the morning because I won’t be able to do anything after taking it. I will just be feeling dizzy the whole day.”* (R25, WLHIV).

#### Emotional health

In terms of emotional and psychological health, there were not much gender differences. All the participants, except two (a MLHIV and WLHIV each), said they were sad, worried and/or distressed about several of the challenges they have.

*“I feel I am just in this world for nothing, I feel hopeless, I don’t have anywhere to go, and I don’t have anything to do …*. *The aspect I don’t like is in relation to my feeding. And also since I was diagnosed, my father does not relate well to me so I am always depressed. I am always not happy with myself. He does not see eye to eye with me … Their relationship with me, they don’t share anything with me. My sisters are siding with my father because they are not HIV patients. So I am alone.”* (R23, WLHIV).

*“Initially when we were supposed to buy the drug, when it’s time to come for your drug and you have no money then you become depressed.”* (R20, WLHIV).

Their worries mostly centred on their financial deprivation and what they felt was seemingly uncertain future for their young dependents: underage children and grandchildren. Furthermore, the majority of the WLHIV worried greatly about their housing challenges. The latter was not the case with any of the MLHIV:

“*Sometimes I think a lot. I am worried and frustrated because I believe anything can happen to me at any time. This is mainly due to financial problems. I think a lot sometimes especially because of my financial crisis.”* (R5, WLHIV, unemployed).

“*… nobody supports me with my children’s school fees and other things. Because of this sometimes I can’t sleep. I am always thinking about some of these issues so I am not happy. Life has not been easy for me at all especially because my husband is dead so I have to take care of all the children by myself.”* (R1, WLHIV, unemployed).

Moreover, most participants, both MLHIV and WLHIV, worried about their HIV/AIDS-positive status:

*“I don’t have any mental problem. The only problem I have is when I start to think of the day this virus will completely leave my system. So that is what I have been thinking about some of the time and when I do that my heart begins to pain me.”* (R29, MLHIV).

*“Because people always say that if you get the virus, you will die. I think deeply until I feel I have thought too much, and stop. I feel disturbed and depressed because people say I will die.”* (R13, WLHIV).

*“You know sometimes you will be there and you begin to think, why should I be sick, why should I always be on drugs, and so on? When you think about those things then you become worried.”* (R30, MLHIV).

The two participants (R15 and R18, WLHIV and MLHIV, respectively), who said they were not worrying about anything at the time of interviewing seemed to have some peculiarities that were mostly different from that of most of the respondents. They were both married, and said their spouses were supportive of them, despite being aware of their HIV-positive statuses; both spouses were HIV-negative. Additionally, both of them were working (a teacher and a petty trader, respectively). Both of them said accessing healthcare, and taking care of their health was not a problem. Among others, they had adequate food, were able to remember to, and do take their medicines regularly and on time, without a problem. They did not need to hide their medicines, and were able to keep their hospital appointments regularly, etc. They both had adequate housing (enough space, rooms, and basic facilities like water, electricity, kitchen, and toilet in their homes). Both also owned their homes (together with their spouses).

Nevertheless, both of them said they had some financial needs, including need for support with accessing healthcare, which is explained mostly by the fact that they both had dependent young children. The MLHIV said some of his siblings regularly support him financially for his healthcare. The MLHIV explained what helps him to stay worry-free:

“[But do you worry or think a lot or are you anxious?] *Oh no not this time. I am old, I am 59 years* [‘59 by November 22nd this year’], *next year I will be 60 years. When I leave the world it’s okay because I read the* [news] *papers and see 41-year-olds, 30-year-olds, who are gone so I am not anxious and not afraid of death.* [Do you sometimes feel depressed?]. *I don’t, because I read a lot of books and the scriptures together. Actually nobody knows my status* [except his wife] *so I am free but if they were to know, there would be places I cannot go; maybe I will have to move from there.”* (R18).

#### Health seeking behaviour

The MLHIV seemed much better resourced to seek healthcare, meet their scheduled hospital appointments, and follow prescribed ART compared to the WLHIV. For instance, more stressful housing and the higher propensity for WLHIV to not disclose to persons within their shared housing, translated into a much higher proportion of WLHIV hiding their medications (ARVs). Due to higher unemployment, the WLHIV were less likely to have money for transportation to the hospital. Furthermore, the MLHIV were getting more help, mostly financial, from their children and siblings, compared with the WLHIV. All participants complained of financial difficulties, including not having money on their own for transportation to the VCTs. Few (one MLHIV and two WLHIV) also mentioned they have difficulties with getting money for purchasing prescribed medications they had to pay out-of-pocket. Furthermore, the two participants who reported that childcare for their young children interferes with attending scheduled hospital appointments were all WLHIV.

*“Because my father is in the family house, if I am coming to the hospital and I don’t have money, I go to him and tell him I am coming to the hospital but I don’t have money and he will give me some. Even today when I was coming he was the one who gave me money to come.”* (R29, MLHIV).

*"Financial problem. I don’t have money so sometimes coming to the hospital is a problem. I have more than four prescriptions from the doctor to buy drugs but I don’t have money so I am unable to have access to the drugs. When drugs are prescribed for me I get only those that are free or those that are covered by the health insurance. Sometimes transportation and what to eat is also a problem. This morning I have only three Ghana Cedis* ($0.75)^1^*on me and owe some money at the dispensary so I won’t be given any drug that is not covered by the health insurance. The one that is not covered by the health insurance, we have to pay five Cedis* ($1.25) *every month so fifteen Cedis ($3.75) for three months so I will not be able to get the drugs today."* (R4, WLHIV).

Furthermore, few participants, both WLHIV (2) and MLHIV (1) complained of conflicts between their work schedules and prescheduled hospital appointments, although they eventually do make up the appointments.

*“Yes, sometimes when you lock up the store and leave to the hospital you go back and find no money for upkeep and since I am not having any savings anywhere, you have to work/sell before we can eat. So if you close the store and you leave what will you eat when you get back?”* (R22, WLHIV).

*“I am a worker and the only problem I face is leaving my work and coming to the hospital. Sometimes I default, if I am told to come on this date I would not be able to come. Today I was late in coming and that has been my only problem. And also when I have to take my drug in the morning I sometimes forget to take it because of work. Also because I have to leave the house early and cannot eat and take my medicines, I forget to take them at work. But the last time I came for drugs I was given a new drug which I have to take only in the night and I think I prefer it that way.”* (R30, MLHIV).

Finally, there were differences in adherence to medication by the MLHIV compared to the WLHIV. This may partly be attributed to the much higher literacy status of the MLHIV compared to the WLHIV. All six participants who were not formally educated were WLHIV. Also, a higher proportion of WLHIV had only basic school education. Only WLHIV mentioned for instance that low level of formal education or the lack thereof, interfered with their ability to take their ARVs on time. In fact, the few participants who mentioned having skipped their previously scheduled hospital appointments or having gone late were WLHIV, except one.

“*I have now been given two months to return. Whiles counting the days, when I see someone I am free with* [an acquaintance] *I ask the person to check the date for me*. *For instance, since I am free with you I will ask you what date this is, and then in two months’ time I will come for it.”* (R34*,* WLHIV, illiterate).

“*I did not go to school so I always show them my card.”* (R31*,* WLHIV*).*

### Challenges with living with HIV/AIDS

#### Stigma, discrimination and abuse

Again, only WLHIV reported being stigmatized, discriminated against or abused. This included being denied food, and/or not sharing family meals with them as mainstream Ghanaian culture dictates, being insulted, accused wrongly, and slapped at the face. Others included family members denying knowing them, shaming, mockery, name calling, and discrimination during attempts to rent a house, and seek employment. Additional forms of stigmatization were constant reminders to some of them that they would die from the infection. The rest included restrictions on their movement within parts of their homes (usually extended family home), such as being restricted to sleep on the veranda, restrictions on using utensils in their homes, restrictions on one WLHIV’s travel, restrictions on the sexual expression of some of them, and picking up quarrels with them without provocation.

*“It is only my father that gives me problems; sometimes he uses words like now that I have grown slim then I am coming to die. I have not told him about it but maybe he heard of it from my sisters because I told them.”* (R5, WLHIV).

*“When the symptoms started … they stopped drinking water from the same pot with me. They stopped eating with me and they told all the kids--those who are not my children but live in the same house with me, not to eat from me.”* (R26, WLHIV).

*“Oh if you are sick! They say I have AIDS, ‘see how it shows all over her body; see how lean she’s grown.’ Because of this sickness I suffer a lot of stigmatization.”* (R34, WLHIV).

One WLHIV said her mother constantly quarreled with her and evicted her from their family home for being HIV-positive.

*“The problem now is my mother, apart from her I don’t have any problem whatsoever with anything or anybody in that house. She is always using my acquiring the virus as an avenue to mock and rain insults on me. We were involved in a heated argument and she said I am not her daughter because all her children do not have the disease so I should leave* [the house]*.”* (R17).

“*I am not allowed to touch anything in the house. Rubber bowls, rubber cups and buckets and other things I am not allowed to touch. And whatever insult they feel like raining on me they do.* [About to cry]. *You will make me weep. As for the insults it’s like the pomade* [body lotion] *I use on my body every day.”* (R23, WLHIV).

“*They discriminate against me. Sometimes, some insult me or say bad things about me when they see me pass by. I become very worried and begin to think again. I sometimes cook but I am not able to eat because of the things people are saying. They scrub the bathroom when only I finish bathing. Some people have warned their children from taking things from me.”* (R1, WLHIV).

The stigma affected some WLHIV’s access to accommodation and employment. 14/32 WLHIV complained of accommodation-related discrimination. One WLHIV mentioned that each time she finished using the sole bathhouse in her compound home, another tenant would scrub it before using it. Two others mentioned that children in their shared homes were barred from interacting with them. One of these mentioned that her daughter had warned her children to not interact with her. WLHIV who lived in rented homes mentioned living in perpetual fear that they would be evicted if their HIV-positive status were found out.

*“In getting accommodation I always pray to get a place where nobody knows about my HIV status … I pray nobody tells them I am HIV-positive else they will change their attitude towards me so all the time I am afraid. If they know you are HIV-positive they will never let you stay in the house because nobody wants to be in the house with such a person.”* (R1, WLHIV).

Almost all WLHIV who did not report stigmatization said they were hiding their HIV-positive status to avoid being stigmatized.

#### Housing/accommodation challenges

Some gender differences in the housing conditions of participants were also observed. These had implications for the health and well-being of the participants. The WLHIV reported much more housing-related problems compared to the MLHIV. The MLHIV lived in more secured housing than the WLHIV. MLHIV were less likely to live in extended family housing premises and had more rooms/space to themselves and their households. Conversely, the majority of the WLHIV were sharing overcrowded extended family accommodations, and were often sharing a single sleeping-cum-living room with several extended and nuclear family members.

Also, WLHIV were more likely to live in rented homes with several tenants. Furthermore, WLHIV were more likely to live in sub-standard accommodations: often without running water, toilet, electricity and kitchen facilities. Other typical housing defects included using temporal structures such as kiosks/quickly-put-together wooden structures as homes. Insecure housing also included homes and their immediate surroundings which were liable to flooding, dusty, weedy, dirty, noisy, or damp. Others were old and dilapidated houses with extensive structural defects or leakages:

*“We are far from the toilet facility and they charge a fee too when you use it. There are places they charge 1 cedi* [$0.25]. *Others also charge 0.50* (12.5 US cents) *pesewas, and the government‘s own is 0.20 pesewas* (5 US cents) *but that place is very far from us. So during emergencies* [to use a toilet] *you cannot go that far.”* (R22).

*“I am not as strong as I used to be so walking long distances just to go to the toilet or the bath is a problem for me.”* (R7, WLHIV).

Furthermore, only WLHIV reported having their homes at insecure places such as muddy, marshy places and locations susceptible to flooding. Few WLHIV reported homes which get flooded when it rains. For more than a half of the WLHIV, such housing limitations interfered with taking their medications. No MLHIV reported housing-related interference with taking their medication.

*“I keep it* [ARV] *in hiding. Staying with someone is not like being on your own.”* (R16, WLHIV).

*“I live in an old family house. Recently the Council* [Municipal Office] *came to warn us that if the building collapses resulting in death, they will not let us go scot-free … the family house is old and so it leaks when it rains. You’d have to collect the water in bowls.* [It is a] *hut* [mud house]. *So even the Council gave us a warning that it could kill someone. But alas! I can’t rent a place … Whenever it’s about to rain, with the winds blowing strongly I become frightened. The roof shakes fiercely. Yes. Just as I said, we set up a bed outside because the room isn’t big. The building is also made with mud, and its state is …* [Frightening?] Y*es, it’s scary, so people from the Council came to warn us. So I am not able to sleep indoors, I sleep outside*.” (R31, WLHIV).

### Employment and financial well-being

Compared to the WLHIV, the MLHIV seemed to fare much better in terms of employment and also, financially. All the participants complained of being needy financially. The fifteen participants who were unemployed comprised one male and fourteen females. Among these, the MLHIV and one WLHIV said the HIV infection had sapped their energy and thus they could not work. Two WLHIV said prospective employers and patrons of their wares discriminate against them and do not employ/buy from them based on their looks; their HIV-positive status was suspected or family members had revealed them to others. Some WLHIV stopped working right after being diagnosed with HIV/AIDS due mostly to self-stigmatization.

All three participants who were formal sector employees were MLHIV. Three participants were trained artisans, comprising two WLHIV who were seamstresses and one MLHIV who was an auto-electrician. Only these two WLHIV out of 18 employed WLHIV had formal training. All the nine petty traders were WLHIV, who did not have any technical training for their vocations. The only MLHIV among the four participants who were farmers was engaged in commercial farming; all the WLHIV were subsistence farmers. With the exception of the MLHIV farmer, all the five MLHIV who were employed had formal training for their jobs. Furthermore, almost all those who stopped working immediately after their HIV-positive diagnosis were WLHIV, although 1/6 MLHIV stopped working lately.

*“Relating my physical health to my work/job I can say I am not able to work effectively anymore. For about four months now I have not been able to do anything, I am just at home. Initially I was a farmer and I was able to work on the farm effectively but since I was diagnosed with this disease I am not able to work anymore. My health is dwindling.”* (R29, MLHIV).

*“If I go to the one who supplies me with the plantain (uncooked staple food) to sell she tells me I am sick to be working so she will sell it herself … I was selling and the person told me ‘Is it you who have HIV who is coming to sell for us to buy so we can also contract HIV? So am I selling so as to share the virus?’ I am searching for work but I am not getting one, they tell me I am too sick to work.”* (R23, WLHIV).

### Food insecurity

Majority (25/38) of the participants mentioned being food insecure. They skipped taking their ARVs because they do not get food/adequate food to eat or do not get food on time for taking the ARVs at the prescribed times. Most of the above-stated deprivation of the WLHIV such as having far less social support and having less secured employment and personal financial resources meant that a higher proportion of WLHIV (23/32) had challenges with food for taking their ARV; 2/6 MLHIV also mentioned having challenges with food.

*“What to eat is also a problem; we have been warned by the nurses not to take the drugs if we don’t eat any good food. Sometimes I don’t take the drugs in the evening because I can’t get good food or enough food to eat due to my financial problem.”* (R7, WLHIV).

“*… it came to a time I was not getting food to eat. I begged for food. That was the reason why I went to buy a flask. My father named the flask ‘beggar’s flask.’ As for the drugs my father forces me to take them but he won’t give me food to eat.”* (R23, WLHIV).

*“What to eat is a problem because some of us are unable to work due to our situation.”* (R4, WLHIV).

These participants mentioned that the food insecurity is a hindrance to taking their ARVs on time or taking it at all. This meant that sometimes, they skipped taking their ARVs due to lack of food:

“*No, you are to eat before taking it at 5:00 but sometimes I don’t eat. But if you don’t eat at 2:00 you cannot take the drug at 5:00; you can’t take the drug at 5:00. Because you can’t take it; it is very strong.”* (R21, WLHIV).

“*… the only barrier* [to taking his ARVS on time] *is when my meal is delayed or I don’t get something to eat. Maybe if I eat twice a day, we were told to eat 3 times a day so if I don’t get food to eat in the morning and I eat around 12 I take my morning drug at 12 and I am unable to take the other one which I am supposed to take at 4:00.* [Interviewer: So sometimes food is a barrier to you when it comes to taking of the drugs.] *Yes because the ladies I am in the house with are young and they don’t have any means of getting food on the table. I am the one supposed to provide the money so if I don’t get it on time then it means the meals will delay.”* (R29, MLHIV).

*“If I get food to eat and I take my medicine I look healthy but if I don’t get food to eat then I feel unhealthy. People ask ‘we just saw you recently and you were looking good how come you have changed?’ So I think if I get good food to eat I will look good … That is what I said that if I don’t take the medicine, it’s because I don’t get food to eat.*” (R23, WLHIV).

## Discussion, conclusion and practice implications

### Discussion

The WHO [10] noted that gender portends health inequities--doing so both singularly, and as a confounding variable to several social indicators such as age, sexual orientation, socio-economic status, ethnicity, and disability. With specific reference to HIV/AIDS, across certain cultures including SSA, the experiences of WLHIV and MLHIV are different [5, 37]. Our findings mostly resonate those from other locations/cultures in SSA. We found that the experiences of our respondent WLHIV were mostly different from those of the MLHIV. In the specific context of the patriachical Krobo culture in the LMKM particularly, and Ghana generally, the differences we found are more cultural than they are biological. Previous researchers [27, 28] noted that the Krobo patrilineal culture gives more social privileges to males compared to females. Others noted the negative effect of culture in translating an HIV-positive status into social problems mostly skewed against WLHIV [5, 7, 37]. Our findings corroborate those of previous authors in SSA [5, 7]. Compared to their female counterparts, the MLHIV this paper studied had higher socio-economic status. Resultantly, they had better command over resources. These translated into giving them privileges over owning and utilizing resources for their betterment. This situation is also generally applicable to the whole of Ghana.

The WLHIV’s assertion that their spouses gave them the infection since the spouses died earlier may explain why our WLHIV had mostly been widowed. Several authors in SSA have noted similarly that for adolescents and younger PLHWAs for instance, men are more likely to infect women [4, 7]. Ramjee and Daniels [5], however, observed that in SSA, WLHIV may have a similar tendency to infect MLHV. Yet in parts of SSA, women are perceived as the transmitters of the HIV virus. The overwhelming heterosexual transmission of HIV in SSA [4, 5] makes society blame women more than men, of sexual-related deviances including adultery and prostitution [5, 37]. Against this background, in mainstream Ghanaian culture, males are permitted to have multiple partners, and to even be polygamists but women are denied such privileges. These behaviours are considered taboo if a woman engages in them [38]. Other studies have generalized such cultural tenets to SSA [4, 5, 7].

Nonetheless, negative branding of PLHWHAs as immoral is often directed towards WLHIV [37, 39]. Consequently, WLHIV are subjected to several inhuman treatment [40]. Undoubtedly, this partly explains why WLHIV in SSA suffer more victimization and discrimination than MLHIV [5, 39], which our study confirmed. Researchers in Iran have reached the same conclusions [37]. Furthermore, as found in this paper, Sia et al. [7] documented higher marital status among MLHIV compared to WLHIV in SSA generally.

Our findings further affirm those of previous authors that poverty disproportionately increases vulnerability to HIV/AIDS, its effects, and consequences on PLWHAs [12, 41]. This is particularly so for women [5, 7]. Poverty also acts through food insecurity, to extend a barrier to some PLWHAs’ treatment for HIV in other parts of SSA [5, 42], as it is in Ghana [43–45]. The result is increased risk for both advancing HIV and onward transmission [46]. The critical link between housing and PLWHAs’ health and well-being even gets worse for WLHIV due to discrimination and gender inequality [13, 14, 47], fuelled by negative cultural practices and beliefs in SSA [40]. Thus, WHO [48] calls for the improvement of women’s human rights and gender equality to reduce the infection rates of HIV for women, and to also improve their access to and uptake of healthcare for HIV.

Similar to our findings, others in SSA found that compared with the MLHIV, WLHIV have much lesser quantity and quality of social support [5, 49, 50]. More interestingly, Vlassoff [51] noted that it is a worldwide phenomenon for males to enjoy more social support. Social integration [52, 53], and belonging support (defined as interaction with friends, family, and other groups) [54] are known to have protective effect on the health and quality of life of some populations with compromised/ailing health status [54]. Our WLHIV participants were mostly separated, divorced or widowed. Prior to this paper’s findings, Sia et al. [7] had explained the importance of the differential distribution of marital status, particularly being widowed, divorced or separated, on the disproportionate distribution of HIV/AIDS in Ghana: unmarried women have twice the risk of HIV compared to unmarried men.

However, aspects of our findings contradict some previous authors. For instance, Girum et al. [55] note that globally, women of reproductive age (15–44 years) are most likely to die from AIDS-related cases. Additionally, our findings that the WLHIV have more difficulty accessing healthcare services regularly compared to the MLIHV deviates from previous research findings that globally, adult women access antiretroviral treatment more than men. This has been attributed to a considerably high coverage [56] and proven effectiveness [57, 58] of prevention of mother-to-child treatment (PMTCT) services for pregnant WLHIV. Moreover, generally, it is explained by women’s use of maternal health services during pregnancy and childbirth. Part of the findings in this paper also contradicted those of Obiri-Yeboah et al. [18] conducted in Ghana: we did not find any MLHIV hiding their ARVs from their wives as they did. Nevertheless, this study found that both WLHIV and MLHIV were hiding their ARVs and healthcare seeking generally.

### Limitations

The key limitation of this study is that it is not possible to draw causal inferences from it because it is not representative of the general population [53]. Resultantly, it is un-generalizable to populations outside the study sample. Another key limitation is the number of MLHIV recruited in the study. Men are generally difficult to recruit in studies [59, 60], which explains the special focus on men during the sampling for this study. However, even with six men, this paper found gendered differences in living experiences of the participants. This probably attests to real differences between the experiences of MLHIV and WLHIV as mostly underscored by the literature.

## Conclusion

This paper undertook a gendered analysis of living with HIV/AIDS in a municipality in the Eastern Region of Ghana, where HIV/AIDS was first found in Ghana [16, 25], and has had the worst impact [23, 25, 29]. The foci was on the SES, health status and healthcare seeking, as well as the daily challenges of the participants. We conclude that gender tremendously influenced the health and well-being of the PLWHAs we studied. The MLHIV were much better off than the WLHIV in several respects. There were marked gender differences in the experiences of the PLWHAs with respect to SES, housing, health status and healthcare seeking, and adherence to prescribed ART. Other differences showed in disclosure of their HIV status, stigma and discrimination, and acceptance by family members and romantic partners. The WLHIV, who were also more likely to have young dependents, were more vulnerable.

Thus, the study revealed that gender negatively filtered the already compromised social status of the PLWHAs we studied, leaving the WLHIV mostly impoverished and at the brink of destitution. The MLHIV were generally better off and faced almost no discrimination, stigmatization and abuse from both within and outside their homes. The paper concludes that rather than focusing on attributing these profound differences in the well-being and experiences of the MLHIV and the WLHIV solely to socio-cultural tenets, these are also human rights issues. We, thus, call for a human rights-based approach to protecting WLHIV from extreme neglect and inhuman treatments/experiences they face due to their gender-filtered HIV-positive status.

The paper affirms WHO’s ([10], p. 1) recommendation that health systems should adopt gender-responsiveness “which acknowledge, understand and transform how gender determines health behaviors, access to services, pathways of health care, and how gender interacts with other determinants of health and drivers of inequities.” Policy makers should work on safeguarding the human rights of WLHIV as well as introduce systems that give WLHIV greater social protection. These should include educating families and community members to pay closer attention to PLWHAs, particularly WLHIV. Increased advocacy should be made for WLHIV. The Ghana AIDS Commission’s and Ghana Health Service’s adverts and public education should highlight the extra burden of HIV/AIDS on WLHIV, and urge the public to empathize with them.

## Data Availability

The datasets used and/or analyzed during the current study are available from the corresponding author on reasonable request.

## Abbreviations

AIDS: Acquired Immune Deficiency Syndrome
ART: Anti-retroviral therapy
COHRE: Center on Housing Rights and Evictions
ECH: Ethics Committee on Humanities
ERC: Ethical Review Committee
GAC: Ghana AIDS Commission
GHS: Ghana Health Service
GNA: Ghana News Agency
HIV: Human immunodeficiency virus
ICAD: International Coalition on AIDS and Development
LMKM: Lower Manya Krobo Municipality
MLHIV: Men living with HIV
NACP: National AIDS Control Program
PI: Principal Investigator
PLWHAs: Persons Living With HIV/AIDS
RA: Research Assistant
SES: Socio-economic status
SSA: Sub-Saharan Africa
WLHIV: Women living with HIV
UNAIDS: United Nations Joint Program on HIV/AIDS
VCT: Voluntary counseling and testing
WHO: World Health Organization

## Footnotes

1 Four Ghana cedis was equal to one United States dollar by the time of data collection
